# Ischemia-reperfusion injury and the risk of hepatocellular carcinoma recurrence after deceased donor liver transplantation

**DOI:** 10.1038/s41598-018-27319-y

**Published:** 2018-06-12

**Authors:** Michał Grąt, Marek Krawczyk, Karolina M. Wronka, Jan Stypułkowski, Zbigniew Lewandowski, Michał Wasilewicz, Piotr Krawczyk, Karolina Grąt, Waldemar Patkowski, Krzysztof Zieniewicz

**Affiliations:** 1 Department of General, Transplant and Liver Surgery, Medical University of Warsaw, Warsaw, Poland; 2 Hepatology and Internal Medicine Unit, Department of General, Transplant and Liver Surgery, Medical University of Warsaw, Warsaw, Poland; 3 Department of Epidemiology and Biostatistics, Medical University of Warsaw, Warsaw, Poland; 4 Second Department of Clinical Radiology, Medical University of Warsaw, Warsaw, Poland

## Abstract

This study aimed to evaluate the effects of ischemia-reperfusion injury (IRI) on the risk of hepatocellular carcinoma (HCC) recurrence after liver transplantation. Data of 195 patients were retrospectively analysed. Post-reperfusion aspartate (AST), alanine transaminase, and lactate dehydrogenase (LDH) levels were the primary measures of IRI. Tumour recurrence was the primary endpoint. Post-reperfusion AST was a continuous risk factor for tumour recurrence in patients within Milan criteria (p = 0.035), with an optimal cut-off of 1896 U/L. Recurrence-free survival of patients within Milan criteria and post-reperfusion AST of <1896 and ≥1896 U/L was 96.6% and 71.9% at 5 and 3.7 years, respectively (p = 0.006). Additionally, post-reperfusion AST and LDH exceeding the upper quartile significantly increased the risk of HCC recurrence in patients within Milan criteria (p = 0.039, hazard ratio [HR] = 5.99 and p = 0.040, HR = 6.08, respectively) and to a lesser extent, in patients within Up-to-7 criteria (p = 0.028, HR = 3.58 and p = 0.039, HR = 3.33, respectively). No other significant IRI effects were found in patients beyond the Up-to-7 criteria and in analyses stratified for independent risk factors for recurrence: tumour number and differentiation, alpha-fetoprotein, and microvascular invasion. Thus, IRI exerts major negative effects on the risk of HCC recurrence after liver transplantation in patients within standard and extended criteria.

## Introduction

Hepatocellular carcinoma (HCC) remains one of the most common indications for liver transplantation^1^. The Milan criteria defined transplant eligibility for HCC patients for more than two decades; however, the limits are now being expanded according to morphological and biological tumour features^2–6^. Nevertheless, discussion on widening the pool of potential candidates is controversial owing to a major and relatively constant shortage of deceased donors. Further expansion of the selection criteria will inevitably lead to increased waiting times for both HCC and non-HCC populations. In HCC patients, markedly prolonged times on the waiting list are characterised by more common dropouts, possibly leading to the development of more aggressive tumours^7^. Owing to increased rates of listing and privileged positions of HCC patients under the current allocation policies, a higher number of HCC transplant candidates may have even more detrimental effects on non-HCC patients’ waiting times and pre-transplant mortality^8,9^. Because widening the donor pool with living donors and high-risk or extended criteria deceased donors is a common strategy, it appears to have major relevance, particularly for HCC patients.

Experimental studies demonstrate the increased risk of cancer recurrence associated with ischemia-reperfusion injury (IRI)^10,11^. Changes in hepatic microenvironment caused by IRI promote seeding and the development of metastases, whereas IRI-induced proinflammatory response, release of growth factors, mobilization of progenitor cells, and transformation of cancer cells to more aggressive phenotypes may potentiate the formation and growth of metastases at both local and remote sites^12–16^. Because grafts procured from high-risk deceased donors and to a lesser extent, partial grafts procured from living donors may be more susceptible to IRI, the use of these grafts may increase the risk of post-transplant HCC recurrence. This hypothesis was subject to numerous studies with inconsistent results. Although transplantations of grafts procured from living donors or high-risk grafts procured from deceased donors after cardiac death or those who were older and had hepatic steatosis or other risk factors were reported to have adverse effects on outcomes after liver transplantations for HCC in several studies, the results of available studies are not completely consistent^17–23^. However, recent reports found that prolonged ischemic times, directly related to the magnitude of IRI, increased the risk of post-transplant HCC recurrence^24,25^. Nevertheless, data on the direct effect of the magnitude of IRI on the risk of HCC recurrence after deceased donor liver transplantation are limited. Therefore, this study aimed to evaluate the association between the degree of graft IRI as indicated by post-reperfusion transaminase and lactate dehydrogenase (LDH) levels and the risk of post-transplant HCC recurrence after deceased donor liver transplantation with respect to patients’ initial risk profile.

## Methods

This was a retrospective observational study. In total, 250 liver transplantations were performed for HCC patients between January 2001 and June 2016 at the Department of General, Transplant and Liver Surgery (Medical University of Warsaw). Patients with fibrolamellar HCCs and those with combined HCC/cholangiocarcinoma were not included. After exclusion of 55 patients with missing measurements of transaminase levels 2 h after reperfusion, the final study cohort comprised 195 liver transplant recipients. The study protocol was approved by the institutional review board of the Medical University of Warsaw. Informed consents were not obtained from the patients due to the retrospective nature of the study, which is in line with institutional review board and national regulations. All methods were performed in accordance with the relevant guidelines and regulations. No organs were procured from prisoners.

The degree of IRI was represented by three variables, namely, serum alanine transaminase (ALT), serum aspartate transaminase (AST), and serum LDH levels; each was assessed from a blood sample obtained 2 h after portal reperfusion. These variables were the primary factors of interest. Peak serum bilirubin concentration, international normalised ratio (INR), and gamma-glutamyl transpeptidase (GGTP) activity over the first 7 post-transplant days were additionally analysed as variables associated with IRI. The duration of cold and warm ischemia was defined as the time from clamping of the donor aorta until the removal of the graft from the preservation solution and that from the removal of the graft from the cold preservation solution until portal reperfusion, respectively. The sum of cold and warm ischemic times formed the total ischemic time. All grafts were procured from donors after brain death. Tumour recurrence over the 5-year post-transplant observation period was the primary end-point. Recurrence-free survival was calculated from the date of transplantation until tumour recurrence and censored at the date of last available follow-up, death for non-HCC related causes or 5 years post-transplantation (whichever occurred first). Details on the surgical technique, perioperative care, immunosuppression, and follow-up protocol are provided elsewhere^26,27^.

First, post-reperfusion ALT, AST, and LDH levels were assessed for their potential effect on the risk of post-transplant tumour recurrence in all patients. Other independent predictors of recurrence were also assessed, including peak post-transplant bilirubin concentration, INR, and GGTP activity. Furthermore, the analyses were adjusted for their potential confounding effects in bivariable analyses. Subgroup analyses were subsequently performed to determine the potential differences in associations between IRI degree and the risk of HCC recurrence according to patients’ initial risk profile, based on fulfilment of selection criteria and established independent predictors of recurrence.

Continuous and categorical variables are given as medians (interquartile ranges) and numbers (percentages). The Kaplan-Meier method was used for survival calculations, and log-rank test was used for intergroup comparisons. A Cox proportional hazards regression analysis was performed to evaluate the associations between particular factors and the risk of recurrence. A multivariable model was created using forward stepwise method with p thresholds of 0.05 and 0.150 for inclusion and exclusion of variables, respectively. An additional series of bivariable analyses were performed to adjust the effects of IRI to potential confounding effects of independent risk factors for tumour recurrence. Spearman correlation coefficients were calculated to evaluate the associations between ischemic times and donor age and post-reperfusion laboratory measurements. Post-reperfusion AST, ALT, and LDH levels; peak post-transplant bilirubin concentration; and peak post-transplant GGTP activity were transformed to their natural logarithms prior to their analyses as continuous variables. Additionally, they were assessed as categorical factors using the upper quartile for division. Receiver operating characteristic (ROC) curves were constructed to determine the optimal cut-offs of continuous factors in predicting recurrence. Hazard ratios (HRs) and c-statistics were presented with 95% confidence intervals (95% CIs). Significance threshold was set to two-tailed p values of 0.05. Analyses were computed in STATISTICA version 13 (Dell Inc., Tulsa, USA) software. The datasets generated and/or analysed during the current study are available from the corresponding author on reasonable request.

## Results

The characteristics of the 195 patients are shown in Table 1. Median AST, ALT, and LDH levels assessed 2 h post-reperfusion were 850 U/L (interquartile range: 486–1625 U/L; range 153–14375 U/L), 566 U/L (interquartile range: 304–935 U/L; range 102–9912 U/L), and 2240 U/L (interquartile range: 1322–4670 U/L; range 385–38207 U/L), respectively. Post-reperfusion AST and ALT levels were significantly, yet poorly correlated, with total (both p = 0.001) and cold ischemic times (both p < 0.001), whereas post-reperfusion LDH levels were poorly correlated with cold ischemic time (p = 0.031), intraoperative fresh frozen plasma transfusions (p = 0.002), and intraoperative packed red blood cell transfusions (p = 0.018, Table 2). Donor age and warm ischemic times were not correlated with post-reperfusion AST, ALT, and LDH levels.

**Table 1:** Recipient, donor, and operative characteristics of 195 liver transplant recipients with hepatocellular carcinoma included in the study.

Variables	Number (%) or median (IQR)
Post-reperfusion AST (U/L)	850 (486–1625)
Post-reperfusion ALT (U/L)	566 (304–935)
Post-reperfusion LDH (U/L)	2240 (1322–4670)
Peak 7-day postoperative bilirubin concentration (mg/dL)	3.6 (2.1–5.6)
Peak 7-day postoperative international normalized ratio	1.5 (1.3–1.8)
Peak 7-day postoperative GGTP activity (U/L)	663 (396–967)
**Recipient sex**	
male	146 (74.9%)
female	49 (25.1%)
Recipient age (years)	58 (52–61)
Hepatitis C virus infection	132 (67.7%)
Hepatitis B virus infection	89 (45.6%)
Model for End-stage Liver Disease	11 (8–13)
Within Milan criteria	113 (57.9%)
Within UCSF criteria	136 (69.7%)
Within Up-to-7 criteria	144 (73.8%)
Number of tumors	1 (1–3)
Diameter of the largest tumor (mm)	30 (20–45)
Total tumor volume (cm^3^)	22 (5–62)
Alpha-fetoprotein concentration (ng/ml)	13.8 (5.7–112.8)
Microvascular invasion	52 (26.7%)
Poor tumor differentiation	19 (9.7%)
Neoadjuvant treatment	102 (52.3%)
Total ischemic time (hours)	9.0 (8.0–10.3)
Cold ischemic time (hours)	8.0 (6.9–9.5)
Warm ischemic time (minutes)	55 (44–68)
Intraoperative PRBC transfusions (units)	3 (1–6)
Intraoperative FFP transfusions (units)	6 (4–9)
Donor age	51 (41–60)
**Donor sex**	
male	120 (61.5%)
female	75 (38.5%)

IQR – interquartile range; AST – aspartate transaminase; ALT – alanine transaminase; LDH – lactate dehydrogenase; UCSF – University of California, San Francisco; PRBC – packed red blood cells; FFP – fresh frozen plasma.

**Table 2:** Analyses of correlations between selected factors and activity of aspartate transaminase (AST), alaninę transaminase (ALT), and lactate dehydrogenase (LDH) at 2 hours after reperfusion in liver transplantation for hepatocellular carcinoma.

	AST activity		ALT activity		LDH activity	
	R	p	R	p	R	p
Total ischemic time	0.241	0.001	0.244	0.001	0.132	0.082
Cold ischemic time	0.324	<0.001	0.312	<0.001	0.188	0.031
Warm ischemic time	0.083	0.324	0.102	0.222	0.124	0.155
Intraoperative PRBC transfusions	0.107	0.143	0.125	0.086	0.179	0.018
Intraoperative FFP transfusions	0.042	0.565	0.055	0.449	0.235	0.002
Donor age	0.010	0.892	0.017	0.815	−0.120	0.108

Correlations were assessed with Spearman correlation coefficients. AST – aspartate transaminase; ALT – alanine transaminase; LDH – lactate dehydrogenase; PRBC – packed red blood cells; FFP – fresh frozen plasma.

**Figure 1:**
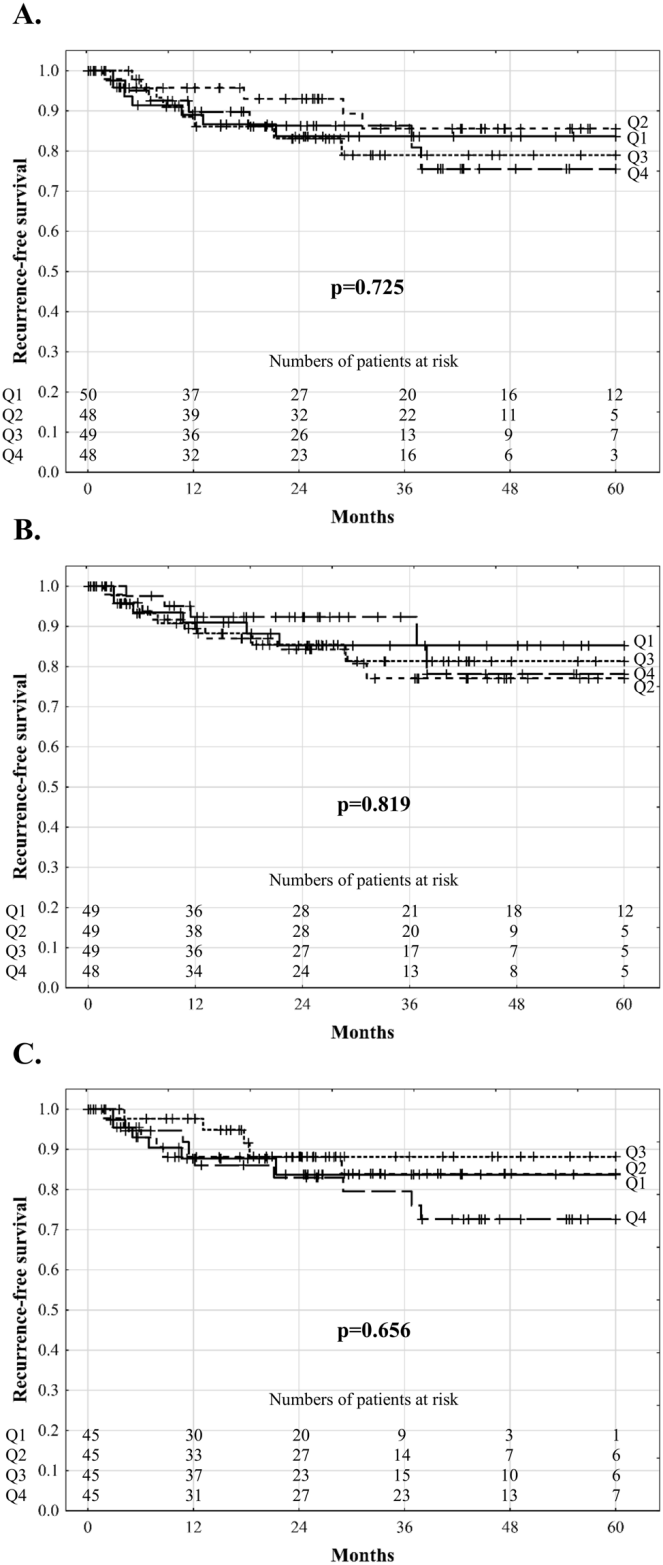
Recurrence-free survival of hepatocellular carcinoma patients after liver transplantation according to quartiles of aspartate transaminase (**A**), alanine transaminase (**B**), and lactate dehydrogenase (**C**) activity 2 hours after portal reperfusion.

**Figure 2:**
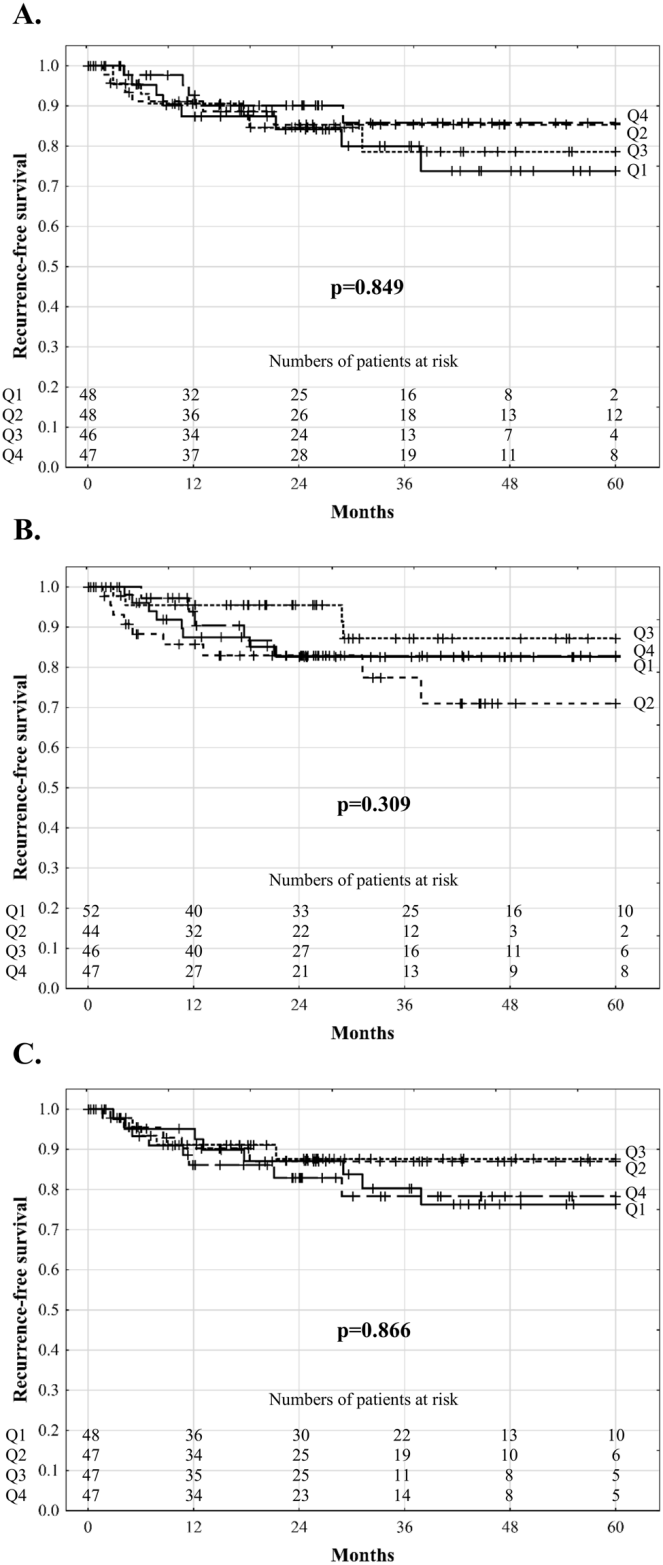
Recurrence-free survival of hepatocellular carcinoma patients after liver transplantation according to quartiles of peak 7-day postoperative bilirubin concentration (**A**), international normalized ratio (**B**), and gamma-glutamyl transpeptidase activity (**C**).

The median follow-up period was 37.5 months. A total of 27 patients developed HCC recurrence with recurrence-free survival rates of 90.8%, 83.4%, and 81.0% at 1, 3, and 5 years, respectively. Univariable analyses revealed that post-reperfusion AST (p = 0.521), ALT (p = 0.773), and LDH (p = 0.575) levels and peak 7-day post-transplant bilirubin concentration (p = 0.592), INR (p = 0.553), and GGTP activity (p = 0.534) were not significantly associated with recurrence in all patients (Table 3). There were also no significant differences in recurrence-free survival depending on the quartile of AST (p = 0.725), ALT (p = 0.819), and LDH (p = 0.656) levels (Fig. 1). Similarly, no differences with respect to recurrence-free survival were observed depending on the quartile of peak 7-day postoperative bilirubin concentration (p = 0.849), INR (p = 0.309), and GGTP activity (p = 0.866; Fig. 2). In multivariable analysis, the independent risk factors comprised tumour number (p = 0.004), pre-transplant alpha-fetoprotein concentration (p < 0.001), presence of microvascular invasion (p = 0.014), and poor tumour differentiation (p = 0.007). No significant effects of post-reperfusion AST (all p > 0.250), ALT (all p > 0.403), and LDH (all p > 0.176) levels and peak 7-day postoperative bilirubin concentration (all p > 0.167), INR (all p > 0.230), and GGTP activity (all p > 0.123) on the risk of recurrence were found in analyses adjusted for the effects of these independent predictors. The corresponding series of bivariable analyses are presented in Tables 4 and 5. Additionally, fulfilment of the Milan (p < 0.001; HR 0.17, 95% CI 0.07–0.43); University of California, San Francisco (UCSF, p = 0.009; HR 0.37, 95% CI 0.17–0.78); and Up-to-7 (p < 0.001; HR 0.24, 95% CI 0.11–0.51) criteria significantly reduced the risk of recurrence.

**Table 3:** Analyses of risk factors for hepatocellular carcinoma recurrence after deceased donor liver transplantation.

Factors	Univariable		Multivariable	
	HR (95% CI)	p	HR (95% CI)	p
Post-reperfusion AST activity (continuous)	1.17 (0.72–1.89)	0.521		
Post-reperfusion AST activity (upper quartile)	1.23 (0.52–2.91)	0.638		
Post-reperfusion ALT activity (continuous)	1.07 (0.67–1.72)	0.773		
Post-reperfusion ALT activity (upper quartile)	0.77 (0.29–2.04)	0.602		
Post-reperfusion LDH activity (continuous)	1.13 (0.73–1.76)	0.575		
Post-reperfusion LDH activity (upper quartile)	1.66 (0.73–3.78)	0.226		
Peak postoperative bilirubin concentration (continuous)	0.86 (0.51–1.47)	0.592		
Peak postoperative bilirubin concentration (upper quartile)	0.67 (0.25–1.78)	0.424		
Peak postoperative INR (continuous)	0.81 (0.41–1.60)	0.553		
Peak postoperative INR (upper quartile)	0.91 (0.34–2.42)	0.854		
Post-reperfusion GGTP activity (continuous)	1.20 (0.67–2.15)	0.534		
Post-reperfusion GGTP activity (upper quartile)	1.36 (0.59–3.12)	0.474		
Total ischemic time	1.02 (0.83–1.24)	0.866		
Cold ischemic time	1.07 (0.85–1.35)	0.544		
Warm ischemic time	1.00 (0.84–1.19)	0.963		
Donor age	1.00 (0.97–1.03)	0.896		
Male donor sex	0.52 (0.24–1.12)	0.095		
Number of tumors	1.25 (1.12–1.41)	<0.001	1.21 (1.06–1.39)	0.004
Diameter of the largest tumor	1.02 (1.01–1.03)	0.001		
Total tumor volume	1.01 (1.00–1.03)	0.170		
Alpha-fetoprotein concentration	1.31 (1.15–1.50)	<0.001	1.29 (1.12–1.47)	<0.001
Microvascular invasion	4.28 (1.99–9.24)	<0.001	2.67 (1.22–5.84)	0.014
Poor tumor differentiation	4.05 (1.71–9.59)	0.002	3.35 (1.38–8.13)	0.007
Neoadjuvant treatment	2.04 (0.91–4.54)	0.082		
Male recipient sex	0.64 (0.29–1.42)	0.269		
Recipient age	1.01 (0.96–1.05)	0.809		
Hepatitis C virus infection	0.89 (0.41–1.95)	0.770		
Hepatitis B virus infection	1.16 (0.54–2.47)	0.703		
Model for End-stage Liver Disease	0.93 (0.84–1.03)	0.186		
Intraoperative PRBC transfusions	0.99 (0.92–1.07)	0.839		
Intraoperative FFP transfusions	0.97 (0.89–1.05)	0.470		

Hazard ratios for continuous variables are given per: 1 log_e_ (U/L) increase for AST, ALT, LDH, and GGTP activity; 1 increase for INR; 1 hour increase for total and cold ischemic times; 10 minute increase for warm ischemic time; 1 year increase for recipient and donor age; 1 increase for tumor number; 1 mm increase for diameter of the largest tumor; 10 cm^3^ increase for total tumor volume; 1 log_e_ (ng/ml) increase for alpha-fetoprotein concentration; 1 point increase for Model for End-stage Liver Disease; and 1 unit increase for transfusions. HR - hazard ratio; 95% CI – 95% confidence interval; AST – aspartate transaminase; ALT – alanine transaminase; GGTP – gamma-glutamyl transpeptidase; INR – international normalized ratio; LDH – lactate dehydrogenase; PRBC – packed red blood cells; FFP – fresh frozen plasma

**Table 4:** Analyses of the effects of the degree of ischemia-reperfusion injury on the risk of hepatocellular carcinoma recurrence after liver transplantation adjusted for the confounding influence of independent risk factors.

Factor	HR	95% CI	p	Adjusted for the effects of:
Post-reperfusion AST activity (continuous)	1.10	0.70–1.74	0.683	Tumor number
Post-reperfusion AST activity (categorical)	1.20	0.51–2.85	0.677	Tumor number
Post-reperfusion AST activity (continuous)	1.11	0.70–1.74	0.667	Alpha-fetoprotein concentration
Post-reperfusion AST activity (categorical)	1.10	0.46–2.62	0.825	Alpha-fetoprotein concentration
Post-reperfusion AST activity (continuous)	1.31	0.76–2.25	0.332	Microvascular invasion
Post-reperfusion AST activity (categorical)	1.67	0.70–3.99	0.250	Microvascular invasion
Post-reperfusion AST activity (continuous)	1.25	0.75–2.09	0.398	Poor tumor differentiation
Post-reperfusion AST activity (categorical)	1.43	0.60–3.41	0.425	Poor tumor differentiation
Post-reperfusion ALT activity (continuous)	1.02	0.64–1.62	0.931	Tumor number
Post-reperfusion ALT activity (categorical)	0.71	0.27–1.87	0.483	Tumor number
Post-reperfusion ALT activity (continuous)	0.96	0.61–1.51	0.856	Alpha-fetoprotein concentration
Post-reperfusion ALT activity (categorical)	0.66	0.25–1.75	0.403	Alpha-fetoprotein concentration
Post-reperfusion ALT activity (continuous)	1.14	0.70–1.87	0.595	Microvascular invasion
Post-reperfusion ALT activity (categorical)	0.90	0.34–2.37	0.827	Microvascular invasion
Post-reperfusion ALT activity (continuous)	1.08	0.65–1.79	0.766	Poor tumor differentiation
Post-reperfusion ALT activity (categorical)	0.89	0.33–2.39	0.824	Poor tumor differentiation
Post-reperfusion LDH activity (continuous)	1.18	0.78–1.81	0.435	Tumor number
Post-reperfusion LDH activity (categorical)	1.69	0.74–3.85	0.211	Tumor number
Post-reperfusion LDH activity (continuous)	1.06	0.69–1.63	0.782	Alpha-fetoprotein concentration
Post-reperfusion LDH activity (categorical)	1.77	0.77–4.06	0.176	Alpha-fetoprotein concentration
Post-reperfusion LDH activity (continuous)	1.06	0.69–1.65	0.784	Microvascular invasion
Post-reperfusion LDH activity (categorical)	1.31	0.57–3.03	0.521	Microvascular invasion
Post-reperfusion LDH activity (continuous)	1.09	0.70–1.69	0.709	Poor tumor differentiation
Post-reperfusion LDH activity (categorical)	1.41	0.61–3.27	0.427	Poor tumor differentiation

HR – hazard ratio; 95% CI – 95% confidence interval; AST – aspartate transaminase; ALT – alanine transaminase; LDH – lactate dehydrogenase.

**Table 5:** Analyses of the associations between peak 7-day postoperative bilirubin concentration, INR value, and GGTP activity and the risk of hepatocellular carcinoma recurrence after liver transplantation adjusted for the confounding influence of independent risk factors.

Factor	HR	95% CI	p	Adjusted for the effects of:
Peak postoperative bilirubin concentration (continuous)	0.78	0.47–1.28	0.324	Tumor number
Peak postoperative bilirubin concentration (categorical)	0.49	0.18–1.35	0.167	Tumor number
Peak postoperative bilirubin concentration (continuous)	0.89	0.54–1.46	0.641	Alpha-fetoprotein concentration
Peak postoperative bilirubin concentration (categorical)	0.69	0.26–1.85	0.465	Alpha-fetoprotein concentration
Peak postoperative bilirubin concentration (continuous)	0.81	0.48–1.39	0.453	Microvascular invasion
Peak postoperative bilirubin concentration (categorical)	0.63	0.24–1.67	0.349	Microvascular invasion
Peak postoperative bilirubin concentration (continuous)	0.83	0.48–1.46	0.526	Poor tumor differentiation
Peak postoperative bilirubin concentration (categorical)	0.71	0.27–1.90	0.500	Poor tumor differentiation
Peak postoperative INR (continuous)	0.64	0.31–1.32	0.230	Tumor number
Peak postoperative INR (categorical)	0.75	0.28–2.01	0.564	Tumor number
Peak postoperative INR (continuous)	0.66	0.33–1.35	0.258	Alpha-fetoprotein concentration
Peak postoperative INR (categorical)	0.77	0.29–2.06	0.603	Alpha-fetoprotein concentration
Peak postoperative INR (continuous)	0.72	0.32–1.60	0.420	Microvascular invasion
Peak postoperative INR (categorical)	0.78	0.29–2.08	0.620	Microvascular invasion
Peak postoperative INR (continuous)	0.86	0.42–1.74	0.673	Poor tumor differentiation
Peak postoperative INR (categorical)	1.23	0.44–3.41	0.696	Poor tumor differentiation
Peak postoperative GGTP activity (continuous)	1.24	0.67–2.29	0.488	Tumor number
Peak postoperative GGTP activity (categorical)	1.42	0.61–3.26	0.415	Tumor number
Peak postoperative GGTP activity (continuous)	1.58	0.83–2.99	0.164	Alpha-fetoprotein concentration
Peak postoperative GGTP activity (categorical)	2.00	0.83–4.84	0.123	Alpha-fetoprotein concentration
Peak postoperative GGTP activity (continuous)	1.28	0.70–2.34	0.422	Microvascular invasion
Peak postoperative GGTP activity (categorical)	1.53	0.66–3.54	0.321	Microvascular invasion
Peak postoperative GGTP activity (continuous)	1.12	0.62–2.01	0.709	Poor tumor differentiation
Peak postoperative GGTP activity (categorical)	1.23	0.53–2.83	0.634	Poor tumor differentiation

Hazard ratios for continuous variables are given per: 1 mg/dL increase for bilirubin concentration; 1 increase for INR; 1 log_e_ (U/L) increase for GGTP activity. HR – hazard ratio; 95% CI – 95% confidence interval; INR – international normalized ratio; GGTP – gamma-glutamyl transpeptidase

For further analyses, the patients were divided into subgroups based on the fulfilment of selection criteria and independent predictors of recurrence. Cut-offs for tumour number of ≥3 and alpha-fetoprotein concentration of ≥48.3 ng/ml were derived from the corresponding ROC curves. As a continuous variable, post-reperfusion AST significantly influenced the risk of HCC recurrence only in patients within the Milan criteria (p = 0.035, Table 6) with the optimal cut-off of ≥1896 U/L. Additionally, post-reperfusion AST and LDH levels exceeding the upper quartiles were significantly associated with increased risk of recurrence in patients either within the Milan (p = 0.039 and p = 0.040, respectively) or Up-to-7 (p = 0.028 and p = 0.039, respectively) criteria. The degree of IRI, as reflected by post-reperfusion AST, ALT, and LDH levels, did not significantly influence the HCC recurrence risk in patients within the UCSF criteria or in those beyond the Milan, UCSF, or Up-to-7 criteria. No other significant associations between post-reperfusion AST, ALT, and LDH levels and the risk of post-transplant tumour recurrence were observed in subgroups derived from divisions based on tumour number, alpha-fetoprotein concentration, presence of microvascular invasion, and degree of tumour differentiation. In contrast to the significant effects of IRI in patients within the Milan or Up-to-7 criteria, no effects were found for the duration of total ischemia (all p > 0.701), cold ischemia (all p > 0.417), warm ischemia (all p > 0.373), and donor age (all p > 0.276) in these subgroups (Table 7). No significant associations between peak 7-day postoperative bilirubin concentration (all p > 0.081), INR (all p > 0.205), and GGTP activity (p > 0.097) and HCC recurrence risk were identified in subgroup analyses (Table 8).

**Table 6:** Subgroup analyses of the associations between post-reperfusion aspartate transaminase, alanine transaminase, and lactate dehydrogenase activity and the risk of hepatocellular carcinoma recurrence after liver transplantation according to fulfillment of selection criteria and independent risk factors.

Factor	Subgroup of patients	Analyzed as continuous variable: per log_e_ (U/L) increase		Analyzed as categorical variable: Q4 versus Q1-Q3	
AST activity	Within Milan criteria	2.75 (1.07–7.03)	0.035	5.99 (1.10–32.78)	0.039
AST activity	Beyond Milan criteria	0.86 (0.47–1.55)	0.606	0.71 (0.21–2.43)	0.591
AST activity	Within UCSF criteria	1.67 (0.84–3.30)	0.141	2.79 (0.94–8.33)	0.065
AST activity	Beyond UCSF criteria	0.84 (0.40–1.75)	0.640	0.36 (0.05–2.77)	0.327
AST activity	Within Up-to-7 criteria	1.91 (0.94–3.90)	0.073	3.58 (1.15–11.11)	0.028
AST activity	Beyond Up-to-7 criteria	0.80 (0.41–1.55)	0.500	0.24 (0.03–1.82)	0.167
AST activity	Tumor number <3	1.58 (0.81–3.09)	0.183	2.22 (0.72–6.80)	0.164
AST activity	Tumor number ≥3	0.81 (0.38–1.70)	0.572	0.62 (0.14–2.79)	0.536
AST activity	AFP < 48.3 ng/ml	1.51 (0.68–3.33)	0.309	1.06 (0.23–5.02)	0.937
AST activity	AFP ≥ 48.3 ng/ml	0.92 (0.50–1.70)	0.801	1.04 (0.37–2.97)	0.936
AST activity	Without MVI	1.37 (0.67–2.80)	0.386	2.58 (0.79–8.47)	0.117
AST activity	With MVI	1.18 (0.51–2.71)	0.694	0.89 (0.20–3.95)	0.882
AST activity	Well or moderately differentiated tumors	1.22 (0.71–2.11)	0.478	1.11 (0.40–3.05)	0.842
AST activity	Poorly differentiated tumors	1.66 (0.34–8.03)	0.526	3.95 (0.75–20.76)	0.104
ALT activity	Within Milan criteria	2.41 (0.88–6.61)	0.087	3.15 (0.63–15.78)	0.163
ALT activity	Beyond Milan criteria	0.84 (0.47–1.50)	0.551	0.43 (0.10–1.84)	0.254
ALT activity	Within UCSF criteria	1.22 (0.62–2.41)	0.571	1.02 (0.28–3.73)	0.974
ALT activity	Beyond UCSF criteria	0.97 (0.48–1.94)	0.924	0.62 (0.14–2.76)	0.527
ALT activity	Within Up-to-7 criteria	1.37 (0.66–2.83)	0.398	1.25 (0.34–4.66)	0.735
ALT activity	Beyond Up-to-7 criteria	0.89 (0.48–1.68)	0.728	0.40 (0.09–1.80)	0.233
ALT activity	Tumor number <3	1.14 (0.58–2.24)	0.699	0.63 (0.14–2.87)	0.555
ALT activity	Tumor number ≥3	0.97 (0.48–1.97)	0.934	0.89 (0.25–3.18)	0.852
ALT activity	AFP < 48.3 ng/ml	1.35 (0.60–3.04)	0.469	0.99 (0.21–4.65)	0.986
ALT activity	AFP ≥ 48.3 ng/ml	0.85 (0.48–1.51)	0.582	0.52 (0.15–1.82)	0.308
ALT activity	Without MVI	1.31 (0.63–2.72)	0.466	1.23 (0.33–4.64)	0.761
ALT activity	With MVI	1.00 (0.52–1.96)	0.989	0.63 (0.14–2.77)	0.540
ALT activity	Well or moderately differentiated tumors	1.02 (0.59–1.75)	0.942	0.79 (0.26–2.37)	0.676
ALT activity	Poorly differentiated tumors	1.67 (0.34–8.13)	0.526	1.80 (0.21–15.23)	0.589
LDH activity	Within Milan criteria	1.93 (0.75–4.97)	0.175	6.08 (1.09–33.95)	0.040
LDH activity	Beyond Milan criteria	0.88 (0.54–1.43)	0.602	0.85 (0.31–2.38)	0.764
LDH activity	Within UCSF criteria	1.33 (0.72–2.45)	0.363	2.75 (0.91–8.27)	0.073
LDH activity	Beyond UCSF criteria	0.90 (0.49–1.66)	0.737	0.74 (0.20–2.77)	0.658
LDH activity	Within Up-to-7 criteria	1.68 (0.86–3.31)	0.130	3.33 (1.06–10.40)	0.039
LDH activity	Beyond Up-to-7 criteria	0.80 (0.47–1.37)	0.420	0.57 (0.15–2.10)	0.398
LDH activity	Tumor number <3	1.21 (0.59–2.45)	0.604	2.33 (0.76–7.15)	0.140
LDH activity	Tumor number ≥ 3	1.04 (0.61–1.76)	0.889	1.03 (0.31–3.44)	0.964
LDH activity	AFP < 48.3 ng/ml	1.16 (0.54–2.52)	0.698	1.66 (0.42–6.67)	0.472
LDH activity	AFP ≥ 48.3 ng/ml	1.01 (0.61–1.68)	0.963	1.49 (0.53–4.14)	0.449
LDH activity	Without MVI	1.15 (0.59–2.27)	0.680	2.13 (0.62–7.35)	0.231
LDH activity	With MVI	0.99 (0.56–1.75)	0.976	0.90 (0.30–2.73)	0.856
LDH activity	Well or moderately differentiated tumors	1.11 (0.66–1.85)	0.703	1.93 (0.76–4.92)	0.169
LDH activity	Poorly differentiated tumors	1.05 (0.43–2.54)	0.922	0.59 (0.10–3.33)	0.547

Q4 – fourth quartile; Q1-Q3 – first to third quartile; AST – aspartate transaminase; ALT – alanine transaminase; LDH – lactate dehydrogenase; UCSF – University of California, San Francisco; AFP – alpha-fetoprotein; MVI – microvascular invasion.

**Table 7:** Analyses of the associations between allograft ischemia and donor age and the risk of hepatocellular carcinoma recurrence after liver transplantation in patient within Milan and Up-to-7 criteria.

Factor	Subgroup of patients	Analyzed as continuous variable:		Analyzed as categorical variable: Q4 versus Q1-Q3	
		HR (95% CI)	p	HR (95% CI)	p
Total ischemia	Within Milan criteria	1.03 (0.69–1.54)	0.893	1.39 (0.26–7.62)	0.701
Total ischemia	Within Up-to-7 criteria	1.05 (0.77–1.41)	0.769	1.01 (0.27–3.72)	0.991
Cold ischemia	Within Milan criteria	1.14 (0.69–1.87)	0.611	2.25 (0.32–16.06)	0.417
Cold ischemia	Within Up-to-7 criteria	1.08 (0.76–1.55)	0.665	1.50 (0.36–6.30)	0.577
Warm ischemia	Within Milan criteria	0.78 (0.42–1.48)	0.452	—^a^	0.373^a^
Warm ischemia	Within Up-to-7 criteria	0.98 (0.70–1.37)	0.908	0.52 (0.06–4.26)	0.544
Donor age	Within Milan criteria	1.04 (0.97–1.13)	0.276	1.71 (0.31–9.54)	0.541
Donor age	Within Up-to-7 criteria	1.01 (0.96–1.06)	0.687	1.03 (0.28–3.84)	0.960

Q4 – fourth quartile; Q1-Q3 – first to third quartile; HR – hazard ratio; 95% CI – 95% confidence interval. Hazard ratios for continuous variables are given per 1 log_e_(U/L) increase. Compared with log-rank test, 100% versus 89.4% recurrence free survival at 5 years in Q4 and Q1–Q3 patients, respectively.

**Table 8:** Subgroup analyses of the associations between peak 7-day postoperative bilirubin concentration, INR value, and GGTP activity and the risk of hepatocellular carcinoma recurrence after liver transplantation according to fulfillment of selection criteria and independent risk factors.

Factor	Subgroup of patients	Analyzed as continuous variable: per log_e_ (U/L) increase		Analyzed as categorical variable: Q4 versus Q1-Q3	
Bilirubin	Within Milan criteria	1.59 (0.50–5.10)	0.436	1.37 (0.25–7.48)	0.717
Bilirubin	Beyond Milan criteria	0.74 (0.41–1.34)	0.318	0.50 (0.15–1.72)	0.275
Bilirubin	Within UCSF criteria	1.23 (0.58–2.65)	0.589	0.95 (0.26–3.46)	0.940
Bilirubin	Beyond UCSF criteria	0.56 (0.26–1.22)	0.142	0.42 (0.09–1.88)	0.254
Bilirubin	Within Up-to-7 criteria	1.56 (0.67–3.61)	0.300	0.98 (0.27–3.63)	0.978
Bilirubin	Beyond Up-to-7 criteria	0.53 (0.26–1.08)	0.081	0.42 (0.09–1.90)	0.263
Bilirubin	Tumor number <3	1.18 (0.55–2.54)	0.666	0.95 (0.26–3.47)	0.942
Bilirubin	Tumor number ≥3	0.59 (0.28–1.24)	0.163	0.42 (0.09–1.89)	0.256
Bilirubin	AFP < 48.3 ng/ml	0.61 (0.26–1.44)	0.262	0.68 (0.14–3.18)	0.620
Bilirubin	AFP ≥ 48.3 ng/ml	1.02 (0.51–2.05)	0.952	0.72 (0.20–2.56)	0.617
Bilirubin	Without MVI	0.62 (0.28–1.41)	0.259	0.31 (0.04–2.43)	0.266
Bilirubin	With MVI	1.00 (0.50–2.01)	0.997	0.87 (0.28–2.72)	0.805
Bilirubin	Well or moderately differentiated tumors	1.01 (0.55–1.86)	0.975	0.73 (0.24–2.20)	0.577
Bilirubin	Poorly differentiated tumors	0.35 (0.08–1.45)	0.149	0.68 (0.08–5.66)	0.717
INR	Within Milan criteria	1.21 (0.23–6.21)	0.823	1.16 (0.13–9.92)	0.894
INR	Beyond Milan criteria	0.58 (0.25–1.35)	0.205	0.55 (0.19–1.66)	0.292
INR	Within UCSF criteria	0.82 (0.25–2.68)	0.747	0.97 (0.21–4.38)	0.968
INR	Beyond UCSF criteria	0.65 (0.25–1.69)	0.380	0.61 (0.17–2.22)	0.452
INR	Within Up-to-7 criteria	0.90 (0.26–3.08)	0.861	1.04 (0.23–4.75)	0.960
INR	Beyond Up-to-7 criteria	0.56 (0.22–1.46)	0.240	0.48 (0.13–1.71)	0.255
INR	Tumor number <3	0.64 (0.14–2.92)	0.569	0.80 (0.18–3.62)	0.774
INR	Tumor number ≥3	0.77 (0.37–1.59)	0.474	0.91 (0.25–3.31)	0.881
INR	AFP < 48.3 ng/ml	1.12 (0.36–3.50)	0.843	1.92 (0.50–7.45)	344
INR	AFP ≥ 48.3 ng/ml	0.53 (0.19–1.48)	0.225	0.39 (0.09–1.73)	0.218
INR	Without MVI	0.38 (0.06–2.52)	0.318	0.46 (0.06–3.63)	0.464
INR	With MVI	0.93 (0.37–2.30)	0.869	1.01 (0.32–3.19)	0.990
INR	Well or moderately differentiated tumors	0.93 (0.48–1.78)	0.817	1.24 (0.45–3.45)	0.678
INR	Poorly differentiated tumors	0.17 (0/01–7.11)	0.351	—	—
GGTP	Within Milan criteria	1.55 (0.48–5.02)	0.467	2.87 (0.58–14.24)	0.196
GGTP	Beyond Milan criteria	1.09 (0.53–2.26)	0.818	1.09 (0.40–3.02)	0.862
GGTP	Within UCSF criteria	1.44 (0.66–3.14)	0.361	1.97 (0.64–6.01)	0.236
GGTP	Beyond UCSF criteria	0.84 (0.32–2.25)	0.732	0.82 (0.22–2.97)	0.758
GGTP	Within Up-to-7 criteria	1.41 (0.61–3.21)	0.420	2.14 (0.68–6.75)	0.193
GGTP	Beyond Up-to-7 criteria	1.00 (0.38–2.60)	0.992	0.86 (0.24–3.11)	0.823
GGTP	Tumor number <3	1.76 (0.77–4.00)	0.177	2.06 (0.67–6.29)	0.207
GGTP	Tumor number ≥3	0.63 (0.25–1.58)	0.321	0.77 (0.21–2.79)	0.689
GGTP	AFP < 48.3 ng/ml	2.47 (0.85–7.21)	0.097	2.40 (0.69–8.28)	0.167
GGTP	AFP ≥ 48.3 ng/ml	1.04 (0.48–2.25)	0.915	1.55 (0.44–5.51)	0.499
GGTP	Without MVI	0.95 (0.41–2.22)	0.910	1.02 (0.27–3.84)	0.979
GGTP	With MVI	1.78 (0.77–4.11)	0.179	2.22 (0.74–6.65)	0.154
GGTP	Well or moderately differentiated tumors	1.36 (0.68–2.69)	0.385	1.47 (0.56–3.88)	0.433
GGTP	Poorly differentiated tumors	0.71 (0.22–2.23)	0.555	0.80 (0.15–4.14)	0.789

Hazard ratios for continuous variables are given per: 1 mg/dL increase for bilirubin concentration; 1 increase for INR; 1 log_e_ (U/L) increase for GGTP activity. Q4 – fourth quartile; Q1-Q3 – first to third quartile; INR – international normalized ratio; GGTP – gamma-glutamyl transpeptidase; AFP – alpha-fetoprotein; MVI – microvascular invasion.

**Figure 3:**
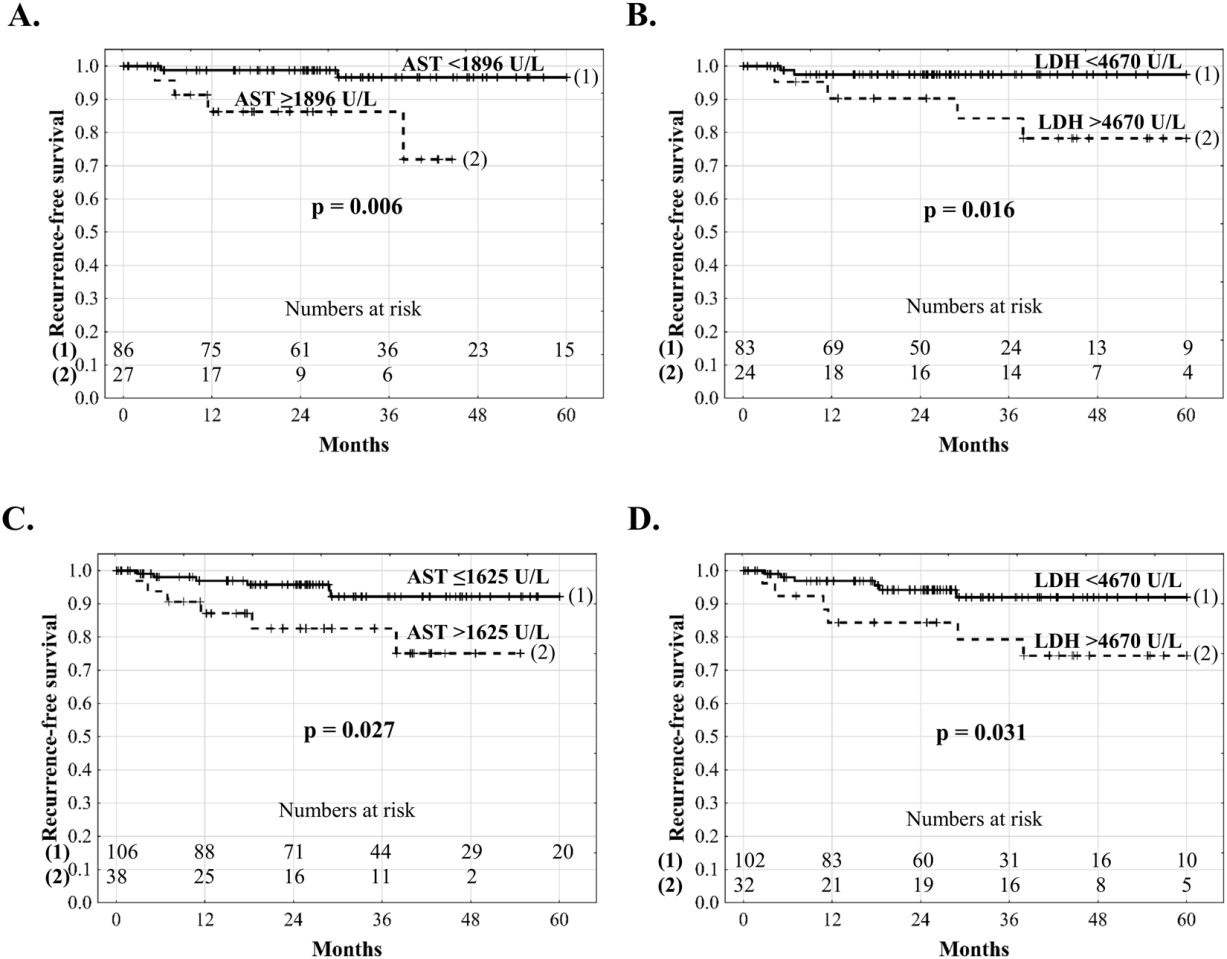
Recurrence-free survival after liver transplantation for hepatocellular carcinoma in patients within Milan criteria (**A**,**B**) and Up-to-7 criteria (**C**,**D**) according to post-reperfusion aspartate transaminase and lactate dehydrogenase activity.

In patients within the Milan criteria, recurrence-free survival at 1, 3, and 5 years was 98.8%, 96.6%, and 96.6%, respectively, when post-reperfusion AST level was <1896 U/L as opposed to 86.2%, 86.2%, and 71.9% at 1, 3, and 3.7 years, respectively, when post-reperfusion AST level was ≥1896 U/L (p = 0.006, Fig. 3A). Similarly, patients within the Milan criteria and with post-reperfusion LDH level <4670 U/L exhibited 5-year recurrence-free survival of 97.4%, which was significantly higher (p = 0.016) than the 1-, 3-, and 5-year rates of 90.2%, 84.2%, and 78.2%, respectively, observed for those within the Milan criteria and with post-reperfusion LDH level ≥4670 U/L (Fig. 3B). Significant differences with respect to 5-year recurrence-free survival depending on post-reperfusion AST (p = 0.027) and LDH (p = 0.031) levels were also observed for patients within the Up-to-7 criteria (Fig. 3C,D).

## Discussion

In the era of donor shortage and increasing utilization of high-risk grafts to partly ameliorate its negative effects, the problem of potential association between the degree of IRI and the risk of HCC recurrence after liver transplantation is of utmost importance. According to the available results of experimental studies, hepatic IRI, universally present in the setting of liver transplantation, increases the risk of metastasis formation both within the ischemic and remote sites through changes in the local microenvironment, induction of inflammatory response, induction of metastatic potential of circulating cancer cells, and systemic release of pro-tumourigenic cytokines^12–16^. Our study results demonstrate a major negative effect of IRI on the risk of post-transplant HCC recurrence, although limited to patients with low tumour burden.

Importantly, initial analyses performed in all patients failed to reveal any significant associations between post-reperfusion AST, ALT, and LDH levels and HCC recurrence risk, irrespective whether the factors were analysed as continuous or categorical variables. However, the study cohort comprised patients with a wide range of tumour burden due to a liberal selection policy utilised in the authors’ department before establishment of precise criteria^5^. Nevertheless, a major significant negative effect of post-reperfusion AST and LDH levels was observed for patients within the Milan criteria, which still determine the majority of liver transplant recipients^28^. Similar findings, although of remarkably lesser extent, were found for patients within the Up-to-7 criteria, whereas the magnitude of IRI did not influence the risk of recurrence in patients beyond the extended criteria. This indicates that the clinical relevance of IRI is limited to generally low-risk populations and diminishes with increasing tumour burden. This appears to be particularly importantly because it demonstrates the possibility of using high-risk grafts to expand the donor pool for high-risk HCC candidates in the context of discussion on widening the boundaries of existing selection criteria^2–6,29^. Notably, the safe use of extended criteria allografts preferentially for patients with advanced tumours was already reported^30^. Conversely, none of the subgroup analyses performed in high-risk patients, including those beyond particular selection criteria, with ≥3 tumours, alpha-fetoprotein concentration ≥48.3 ng/mL, or with tumours either poorly differentiated or with microvascular invasion, revealed a significant effect of IRI on the risk of HCC recurrence. Therefore, while these findings point toward the possibility of the utilization of grafts more prone to IRI for high-risk HCC patients, they also indicate limited clinical relevance of reducing IRI in these patients.

In contrast to the use of post-reperfusion transaminases and LDH levels as surrogates of IRI degree in the present study, previous studies focused on the negative effects of prolonged graft ischemia or donor characteristics^17–25,31^. However, the degree of IRI is driven by the interplay of several donor risk factors, of which a single component may not necessarily be an adequate measure of IRI^32^. In the present study, the laboratory measures of graft ischemia were significantly, yet poorly correlated to graft ischemic times, which in fact is consistent with the results presented by other authors^25^. This may partly explain the inconsistent results of studies on the effect of duration of graft ischemia and particular donor factors on HCC recurrence risk, as these may not always accurately reflect the magnitude of IRI^17–25,31^.

In contrast to the significant effects of IRI limited to low-risk patients found in the present study, two previous analyses specifically aimed at the effect of ischemic times on tumour recurrence revealed the presence of significant associations particularly in high-risk HCC patients^24,25^. These populations were characterised by ^18^F-fluorodeoxyglucose tumour avidness on pre-transplant positron emission tomography and vascular invasion, both of which are known surrogates of biological aggressiveness. Although positron emission tomography data were not available, categorization of patients based on pre-transplant alpha-fetoprotein concentration and tumour differentiation, which are important markers of tumour biology, did not reveal any significant effects of IRI and neither did the analyses stratified for microvascular invasion. The reason for this discrepancy is unclear, although it may be related to a wider spectrum of tumour burden in patients included in the present study. Of note, post-operative peak transaminases did not emerge as risk factors for HCC recurrence in these previous reports. However, we chose post-reperfusion AST, ALT, and LDH levels routinely assessed in our department and not peak levels over the postoperative period in order to minimise the effect of events other than IRI on these parameters.

The results of the present study point toward the importance of strategies aimed to decrease IRI particularly for patients within the standard selection criteria. A single retrospective study revealed decreased magnitude of IRI, as illustrated by low transaminase levels and decreased risk of HCC recurrence in patients receiving prostaglandin E1 analog alprostadil in the early period after liver transplantation^33^. The protective effects of ischemic preconditioning with respect to the development of metastases were also reported in a recent experimental study^14^. The use of machine perfusion devices has also been shown to decrease the magnitude of IRI and recently even enabled the development of a strategy to practically eliminate its negative consequences^34–36^. Although the present study does not provide any evidence for the effects of these measures in liver transplantation for HCC, it provides a rationale for prospective trials aimed at addressing this issue.

This study had several limitations besides those inherent to its retrospective nature. Donor characteristics other than a baseline variable of age were neither analysed for associations with post-reperfusion transaminase and LDH levels nor as predictors of tumour recurrence. However, such analyses were beyond the scope of this study, specifically aimed at the effect of IRI on post-transplant HCC recurrence rather than on its determinants. Because all recipients received grafts from donors after brain death, this study did not directly address the issue of using grafts from donors after cardiac death for HCC patients, which was recently shown not to increase the risk of post-transplant recurrence^23^. Although subject to additional warm ischemia and thus potentially increased magnitude of IRI, their use in HCC patients may be confounded by other factors, including but not limited to, non-random allocation and differences in other donor characteristics. Furthermore, the duration of warm ischemia was not identified as a significant predictor of HCC recurrence. Finally, the main findings of our study are based on the results of univariable subgroup analyses. Therefore, the findings may be confounded by the effects of other risk factors for tumour recurrence. Although there was no particular policy at the authors’ department for the allocation of high-risk grafts to higher-risk HCC patients, the results may also be confounded by non-random allocation of grafts more prone to IRI to patients within the Milan or Up-to-7 criteria, yet at higher initial recurrence risk.

In conclusion, the magnitude of IRI is strongly associated with the risk of tumour recurrence in patients within the Milan criteria and to a lesser extent, in patients within the extended criteria. Available measures to decrease IRI should be evaluated as a method to prevent HCC recurrence after liver transplantation, specifically in patients with low tumour burden.
